# Structure and Mechanical Properties of AlMgSi(Cu) Extrudates Straightened with Dynamic Deformation

**DOI:** 10.3390/ma17163983

**Published:** 2024-08-10

**Authors:** Dariusz Leśniak, Józef Zasadziński, Wojciech Libura, Beata Leszczyńska-Madej, Marek Bogusz, Tomasz Latos, Bartłomiej Płonka

**Affiliations:** 1 Faculty of Non-Ferrous Metals, AGH University of Krakow, 30-059 Kraków, Poland; zas@agh.edu.pl (J.Z.); libura@agh.edu.pl (W.L.); bleszcz@agh.edu.pl (B.L.-M.) bogusz@agh.edu.pl (M.B.); tomaszlatos@interia.pl (T.L.); 2 Łukasiewicz Research Network—Institute of Non-Ferrous Metals, 32-050 Skawina, Poland; bartlomiej.plonka@imn.lukasiewicz.gov.pl

**Keywords:** AlMgSi(Cu) alloys, extrusion, straightening, dynamic deformation, microstructure, mechanical properties

## Abstract

Before artificial ageing, extruded aluminium profiles are subjected to stretching with a small cold deformation in the range of 0.5–2%. This deformation improves the geometrical stability of the extruded product and causes changes in the microstructure of the profile, which leads to the strain hardening of the material after artificial ageing. The work has resulted in the creation of the prototype of an original device, which is unique in the world, for the dynamic stretching of the extruded profiles after quenching. The semi-industrial unit is equipped with a hydraulic system for stretching and a pneumatic system for cold dynamic deformation. The aim of this research paper is to produce advantageous microstructural changes and increase the strength properties of the extruded material. The solution of the dynamic stretching of the profiles after extrusion is a great challenge and an innovation not yet practised. The paper presents the results of microstructural and mechanical investigations carried out on extruded AlMgSi(Cu) alloys quenched on the run-out table of the press, dynamically stretched under different conditions, and artificially aged for T5 temper. Different stretching conditions were applied: a static deformation of 0.5% at a speed of 0.02 m/s, and dynamic deformation of 0.25%, 0.5%, 1%, and 1.5% at speeds of 0.05 and 2 m/s. After the thermomechanical treatment of the profiles, microstructural observations were carried out using an optical microscope (OM) and a scanning electron microscope (SEM). A tensile test was also carried out on the specimens stretched under different conditions. In all the cases, the dynamically stretched profiles showed higher strength properties, especially those deformed at a higher speed of 2 m/s, where the increase in UTS was observed in the range of 7–18% compared to the classical (static) stretching. The microstructure of the dynamically stretched profiles is more homogeneous with a high proportion of fine dispersoids.

## 1. Introduction

After hot extrusion and quenching, aluminium alloy profiles are usually subjected to a stretching process on the run-out table of the press. The stretching process is carried out at room temperature when it is safe to handle the material.

In fact, stretching is part of the thermomechanical processing of the extruded profiles, which consists of three steps: quenching on the run-out table of the press, cold tension in the stretcher, and ageing. Cold forming, e.g., by stretching, plays an important role in this process. A small static tensile deformation in the range of 0.5–2% is applied to straighten the profile, which reduces the residual stress level within the product and causes additional changes within the material microstructure. These changes enhance the subsequent ageing process, and ultimately result in the strengthening of the profile.

In his work [1], Kazanowski investigated the effect of various process and material parameters on the stretching of a profile. He also evaluated the theory of material deformation at the different stages of the stretching process.

Many papers deal with the influence of stretching, also referred to as pre-stretching, on the behaviour of aluminium alloys during thermomechanical treatment. Furu et al. [2] investigated the effect of pre-stretching on the aluminium alloys AA6005, AA6060, and AA6082. Rectangular profiles were extruded and pre-stretched with the plastic strains of 1.0%, 5.0%, and 10.0% before artificial ageing. For all three alloys, the experimental results showed a difference in yield strength for different levels of pre-stretching. For AA6005 pre-stretched 10.0%, the yield strength was approximately twice that of the unstretched sample in the underaged condition.

The effect of pre-stretching on plasticity and fracture was investigated by Abi-Akl and Mohr for AA6451 aluminium sheets [3]. The selected specimens were pre-stretched at 2.0% and 5.0%, followed by artificial ageing at 180 °C for 20 min for all the specimens. Uniaxial tensile tests showed that a higher degree of pre-stretching resulted in a higher yield strength.

In [4], the effect of pre-stretching on the mechanical behaviour of extruded rectangular hollow profiles made from three aluminium alloys, namely AA6063, AA6061, and AA6110, was investigated. Either 0.5% or 4.0% pre-stretch was applied after extrusion and before artificial ageing to temper T6. The uniaxial tensile tests showed that the 4.0% pre-stretched alloys exhibited significantly better ductility than the 0.5% pre-stretched alloys. The improved ductility was also obtained in the crush tests of the profiles, where the 4.0% pre-stretched alloys showed fewer cracks than the 5.0% pre-stretched alloys.

Qvale et al. [5] investigated the effect of pre-stretching on the microstructure and mechanical behaviour of the extruded profiles of the aluminium alloys AA6063 and AA6082. The profiles were pre-stretched at 0.5% and 4.0% after extrusion and prior to artificial ageing to T6 temper. The uniaxial tensile tests showed a higher yield strength for AA6063 and a lower yield strength for AA6082 in the 4.0% pre-stretched condition compared to the 0.5% pre-stretched alloys.

Wang et al. [6] investigated a novel thermomechanical treatment that improved the strength of an AA6061 aluminium alloy sheet. The main steps in the process included under-ageing, cold rolling, and re-ageing, where they were able to achieve a yield strength of 542 MPa and a UTS of 560 MPa for the alloy. The increased strength was the result of the large amount of cold work applied (75% reduction in sheet thickness). However, this level of cold work is not achievable for extrusions that have been stretched prior to artificial ageing.

Kolar et al. [7] studied alloy 6060 subjected to various degrees of pre-stretching between 0 and 10% followed by artificial ageing at 190 °C for between 10 and 300 min. The results showed that the yield stress and UTS improved with the increasing levels of pre-stretching, with the greatest effect at the shorter ageing times.

Ma and Robson [8] investigated a strategy to achieve ultra-high strength for the 7075 alloy by combining work hardening and precipitation strengthening. To achieve this, they carried out experiments consisting of solution heat treatment, pre-ageing, deformation, and second ageing. The results showed that uniaxial deformation to a 10% strain had little effect on precipitates but introduced sufficient dislocations to cause significant work hardening resulting in strength greater than that obtained with a T6 temper.

The influence of plastic deformation prior to artificial ageing on the microstructure evolution and mechanical properties of a novel Al–Li–Cu–X alloy was investigated in [9,10]. The plastic deformation ranged from non-stretched to 8% stretch, with intermediate stretches of 2%, 4%, and 6%. The pre-age deformation improves the ageing kinetics, number density, and strength of fine precipitates by introducing heterogeneous matrix nucleation sites. Increasing the pre-strain level to 15% resulted in an increase in the T8 yield strength to ∼670 MPa with a reduction in ductility from ∼11 to 7.5% [10].

Zuo et al. [11] studied the effect of creep strain, mechanical properties, and microstructure of the 7055 alloy under different pre-stretch conditions. The results show that the range of pre-stretch from 1.6% to 3.3% is suitable for the creep-aged 7055-T6 alloy to obtain better mechanical properties.

Yang et al. [12] investigated the effects of pre-deformation on the creep strain, mechanical properties, and microstructures of the AA2219 aluminium alloy. The pre-deformation can prolong the duration of the primary creep stage and facilitate the creep strain. The effects of 0~9% stretching on the microstructure and mechanical properties were investigated in Al-Li-Cu-Mg alloys [13]. Stretching made T(AlCuLi) precipitates finer and more uniform. Stretching at 6% improved the yield strength of the aged alloy from 328~342 MPa to 466~488 MPa, but reduced the elongation from 9.7~10.4% to 5.7%.

In [14], the influence of pre-straining and pre-ageing on the precipitation behaviour and age-hardening response for Al-Mg-Si alloys was investigated. The authors found that the dislocations introduced by the pre-strain treatment enhanced the inhibition of natural ageing and hardening at room temperature. The dislocations provided heterogeneous nucleation for the precipitates that formed the β″ strengthening phase during the bake hardening treatment. The age-hardening response of the Al-Mg-Si alloys improved with the formation of the denser and larger β″ strengthening phase.

In [15], the effects of pre-stretching and various ageing treatments on the tensile properties and fracture toughness of the 7050 aluminium alloy were studied. The results showed that the peak-aged alloy had higher strength but poor fracture toughness.

Quan et al. [16] investigated the effect of pre-stretching after solution treatment on the hardness and microstructure of aged 2524 aluminium alloy. The results showed that compared with the unstretched samples, the hardness increased and the time to reach the peak hardness decreased with increasing pre-strain. The number density of S (Al_2_CuMg) phases was increased and the length was shortened in the pre-stretched alloy.

Yu et al. [17] presented a new pre-deformation treatment method for the 2324 aluminium alloy. The treatment method combined cold rolling deformation and pre-stretching deformation which increased the crystal defects. A large number of dislocation networks and tangles significantly improved the strength of the alloy and its microstructure.

Similar results were obtained for the 2219 alloy by Liu et al. in [18]. They investigated the influence of 2% pre-deformation on the ageing process.

The influence of pre-stretching on the mechanical properties of 2219 Al alloy sheets was investigated in [19]. The introduction of pre-stretching resulted in an increase in yield strength. The peak yield strengths of 387.5 and 376.8 MPa are obtained when the specimens pre-stretched by 10% are aged at 150 and 170 °C, respectively, which are higher than those obtained for the unstretched specimens (319.2 MPa).

Taichman et al. [20] studied the effect of deformation prior to ageing on the precipitate microstructure and precipitate types in an undeformed and a 10 pct pre-deformed condition of the commercial AA6060 alloy. The tensile tests showed that the yield strength was higher with pre-deformation for different ageing times.

The above publications show that the stretching process followed by artificial ageing has a complex effect on the microstructure. Pre-stretching creates dislocations which become heterogeneous nucleation sites for precipitates during the subsequent ageing.

Łatkowski carried out comprehensive investigations on the thermomechanical treatment of aluminium alloys [21]. He applied plastic strains of 0%, 5%, 10, and 15% to alloy 6060 before artificial ageing. The hardness increased to about 77 HB as the pre-ageing deformation increased up to 15%. For the 2017 alloy, after 24 h of natural ageing and 8 h of artificial ageing, he obtained the yield strength and UTS of about 650 MPa and 670 MPa, respectively.

Research on the addition of various other elements, such as Ni, Co, Au, and Cd to 6xxx alloys is reported in the review paper [22] to summarise the influence of these elements on the evolution of microstructure and its correlation with mechanical properties. Cu alters the precipitation sequences in these alloys and produces a variety of precipitates, resulting in the improved strength properties of the alloys.

It is clear from the papers cited that static pre-deformation prior to ageing improves the strength properties of the alloys tested. On the other hand, some studies reported in the literature indicate a very promising possibility of using the dynamic deformation of the material prior to ageing. Such deformation of about 1–1.5% produces a dislocation microstructure which improves the strength properties of the alloys after ageing.

The yield strengths obtained from dynamic loading at a strain rate of 4000 s^−1^ are 17% and 19% higher than those obtained from quasi-static compression for the T6 and HT specimens of 6061 alloy, respectively [23]. The higher initial dislocation density in T6 specimens hinders the movement of newly formed dislocations and therefore increases the strength of the material.

The influence of a novel thermomechanical process route on the microstructural evolution and dynamic tensile deformation behaviour of two aluminium alloys, i.e., AA6082 and AA7075, was investigated by Sharifi et al. [24]. Dynamic tensile tests were carried out at strain rates of 40, 200, and 400 s^−1^. As the strain rate increased, the strength and elongation to failure of the alloys investigated increased for all the conditions.

The work of Tong et al. [25], which is not directly applicable to the extrusion process, explains to some extent the influence of dynamic stretching on the strengthening effect in the aluminium alloys subjected to thermomechanical treatment. During dynamic stretching, the deformation time is short and there is not enough time for dislocations to undergo annihilation and rearrangement processes. The dynamic recovery process is strongly inhibited as the higher density of dislocations is maintained and the uniformity of the dislocation distributions is improved. As the strain rate increases, the speed of dislocation movement also increases, resulting in a significant increase in peak strength.

Based on the data on the influence of dynamic deformation on the microstructure and mechanical properties of aluminium alloys, it can be concluded that even a small deformation of 1–1.5%, as usually used in the stretching of an extruded profile, can produce a microstructural effect equivalent to about 10% of the static deformation.

In the present work, the authors have built a prototype device for the dynamic stretching of extruded profiles after quenching. The semi-industrial device is equipped with a hydraulic system for stretching and a pneumatic system for cold dynamic deformation. The aim of this project is to produce favourable microstructural changes and increase the strength properties of the extruded material.

The microstructure and mechanical properties of the profiles were studied on extruded AlMgSi(Cu) alloys, which were water-quenched on the run-out table of the press, and subjected to static and dynamic stretching before artificial ageing to T5 temper.

## 2. Materials and Methods

### 2.1. Characterisation of AlMgSi(Cu) Alloys

Billets with the chemical composition shown in Table 1 and a diameter of 100 mm were direct-chill (DC) cast under semi-industrial conditions. Three alloys within the AlMgSi(Cu) grade were investigated. In all the alloys studied, the low-melting microstructural components were dissolved during homogenisation soaking to a degree sufficient for practical use—no incipient melting peaks were observed on the differential scanning calorimetry (DSC) curves. As a result, a significant increase in the solidus temperature was achieved, and the values obtained ranged from 574.6 °C for alloy 3 to 596.1 °C for alloy 1 (Table 2).

### 2.2. Device for Dynamic Straightening

**Figure 1:**
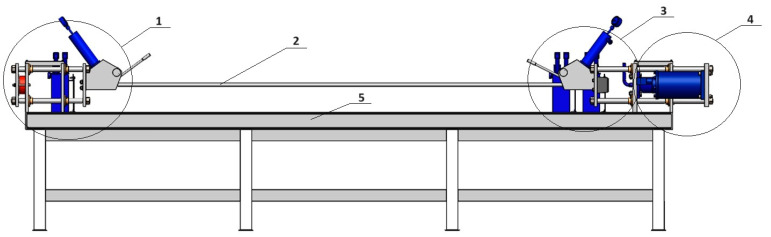
Device for the cold dynamic stretching of the extruded profiles from the AlMgSi(Cu) alloys, view from the face: 1—clamp jaw with elastomer; 2—aluminium profile; 3—clamp jaw on the right side; 4—dynamic force system; 5—pedestal.

**Figure 2:**
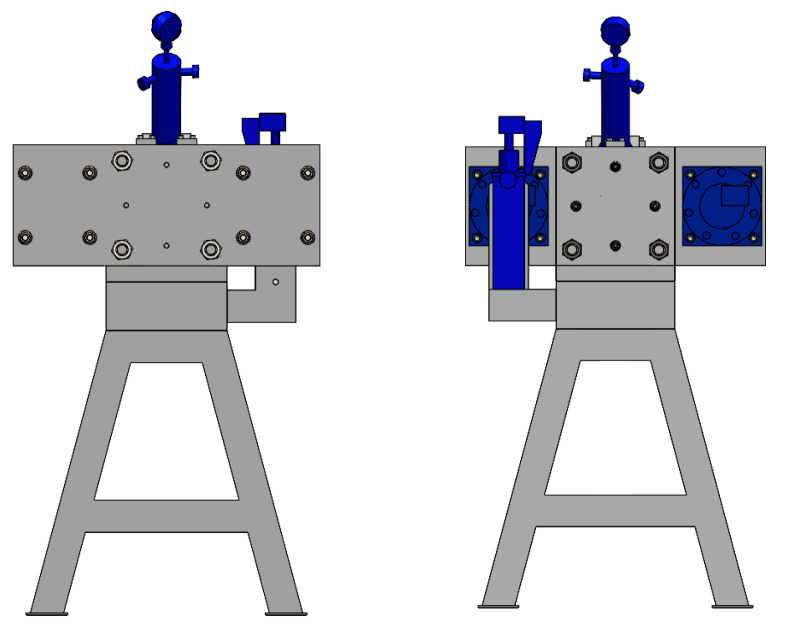
Device for the cold dynamic stretching of the extruded profiles from the AlMgSiCu) alloys, view from the back and front.

**Figure 3:**
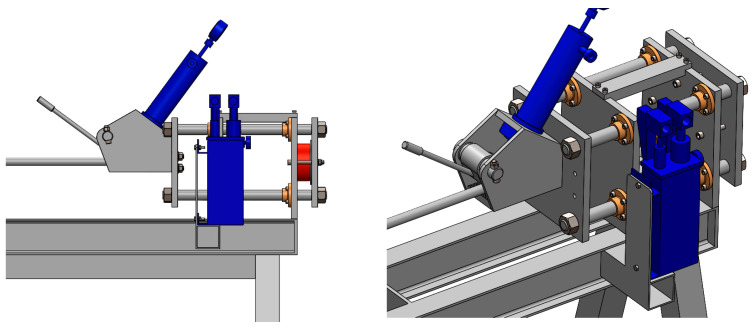
Device for the cold dynamic stretching of the extruded profiles from the AlMgSi(Cu) alloys, view from the right side of the system.

In order to add the effect of dynamic deformation to the static stretching of the extruded aluminium profiles, a semi-industrial device was designed and equipped with a dedicated dynamic system. The system stretches the profile statically by means of a hydraulic cylinder and simultaneously applies the dynamic effect in the form of cyclic impacts of the special dynamic hammers. The maximum stretching force is assumed to be as high as 40–60 kN. The structure of the device is based on a solid assembly table and two heads for the profiles (left and right). The two heads equipped with jaws are movable with respect to the table, one of them being able to move by pulling force only with a constant force parameter mediated by an elastomer suppression system, while the other one moves with the increase in the pulling force and with the deformation of the profile. Figure 1 shows the profile view of the entire workstation; Figure 2 shows the view from the left and right sides (back and front). Figure 3 shows the right side of the set, where a system for maintaining the constant tension of the profile is located. The system is based on the application of a special elastic element between the two steel plates that close when the pulling force is applied. The two plates move on the four sliding columns positioned vertically with respect to the assembly table. On the leftmost plate, there is a jaw for clamping the profile, which is equipped with a hydraulic cylinder to generate the correct clamping force. The hardness of the elastic element must be calculated on the basis of the section and condition of the profile being tested in order to ensure that the system operates within its optimum force parameters.

**Figure 4:**
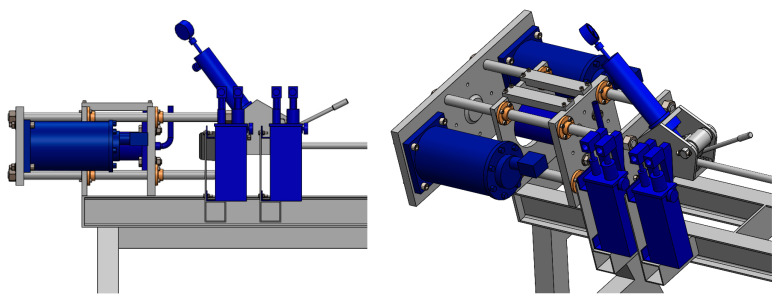
Device for the cold dynamic stretching of the extruded profiles from the AlMgSi(Cu) alloys, view from the left side.

Figure 4 shows the left side of the dynamic system, where the hydraulic cylinder is mounted to generate the static force and the set of two synchronised pneumatic hammers to generate the additional dynamic forces. The two moving plates are also located on this side. These two plates can be moved on the four sliding columns that are perpendicular to the assembly table. The jaw is mounted on the lower plate to hold the profile, similar to the one on the right side of the system. The dynamic set is mounted on the larger plate. In addition, the larger plate is pushed by the 50 T hydraulic cylinder on the vertical axis to generate the stretching force. The number of cycles and the duration of the impact are programmable and must be adapted to the given profile on the basis of the experimental data and its initial characteristics.

### 2.3. Extrusion Trials and Straightening Process

**Figure 5:**
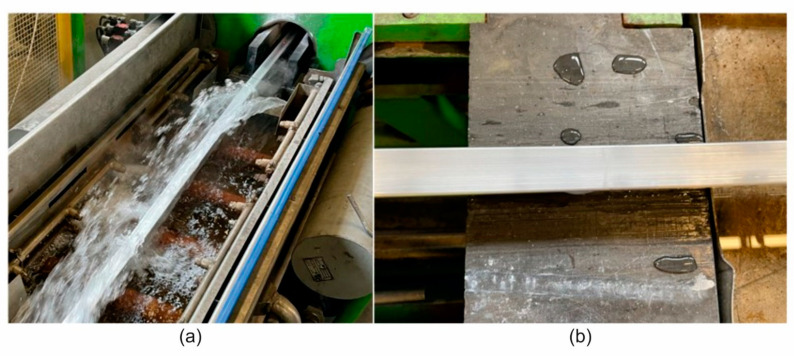
The 5 MN 4-inch extrusion press run-out table with water wave installation (**a**) and the profile from AlMgSi(Cu) alloy after extrusion with cooling by water on the run-out table (**b**).

The extrusion trials for the 60 × 40 × 2 mm (alloy 1 and alloy 2) and 50 × 30 × 3 mm (alloy 3) from the AlMgSi(Cu) alloys using the porthole dies were carried out on a 5 NM hydraulic direct press equipped with a 4” diameter container and water wave installation (Figure 5a). The following process parameters were recorded during the trials: a metal exit speed of approximately 10 m/min, extrusion force of approximately 4.5 MN, and profile temperature of approximately 540 °C using the data acquisition system. The surface quality was checked online for cracks or tarnishing. The maximum extrusion speed applied causes cracks to appear on the surface of the extrudates. The samples taken from the profiles extruded under different process conditions were subjected to optical scanning to check their dimensional accuracy. Similar optical scanning was carried out on the used dies and their dimensions were compared with those of the new dies.

**Figure 6:**
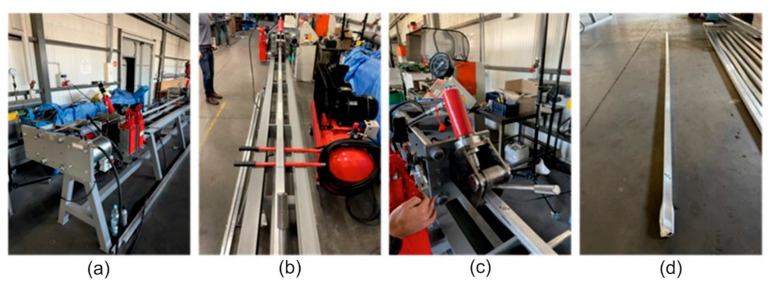
The prototype device for the dynamic stretching of the extruded profiles from the AlMgSi(Cu) alloys—the photos from the experimental tests: (**a**) side view, (**b**) front view, (**c**) the close-up view of the clamp jaw and dynamic force system and (**d**) the sample of the extruded profile after the dynamic straightening.

The dynamic stretching tests were carried out with the prototype device on the water-quenched profiles of the following dimensions: 60 × 40 × 2 mm (alloy 1A and 2A) and 50 × 30 × 3 mm (alloy 3A). The dynamic deformation during the test was increased to the level of 0.25%, 0.5%, 1%, 1.5%, and 2% at stretching speeds of 0.05 and 2 m/s. For comparison, static stretching was also performed for a deformation of 0.5% and at a stretching speed of 0.02 m/s. Figure 6 shows the photographs of the test using the prototype device. After the static and dynamic stretching, the profiles were subjected to artificial ageing at a temperature of T = 175 °C and for an ageing time of 8 h.

The aluminium profiles used in the tests were 3600 mm long. The profiles are mounted on both sides of the system in the hydraulic jaws of 25 T capacity. After clamping, an initial tension of up to 50% of the force required for the plastic deformation of the profile is applied. The profile is then stretched by means of a hydraulic cylinder with a maximum force of 50 T to achieve plastic deformation. At the same time, the pneumatic hammers synchronously strike the main plate on the left side to generate an additional deformation by the dynamic shock wave. The number of impacts and repeatable cycles depends on the test assumptions and the final result for the given profile. During the impact of the pneumatic hammers, the elongation of the profile occurs, and the constant force maintenance system keeps the device under tension all the time. When the correct deformation is obtained, as read on the straightedge, the profile is removed from the jaws. The device is now ready for the next test.

All the conditions of the extrusion process of the profiles, extrudate stretching, and ageing were shown in Table 3.

### 2.4. Methodology of the Microstructural and Mechanical Examination

The samples for microscopic examination were mounted in resin, mechanically ground with sandpaper of appropriate grit, and then mechanically polished in two stages using a diamond paste suspension and a colloidal silica oxide suspension for the final polishing. In order to reveal the microstructure of the samples for observation by light microscopy, the samples were anodised in a Barker reagent with a composition of 100 mL H_2_O + 2 mL HBF_4_. The microstructure of the samples was examined by light microscopy (OLYMPUS GX51 microscope, Tokyo, Japan) and scanning electron microscopy (Hitachi SU 70 microscope, Tokyo, Japan). In addition, the chemical composition within the micro-areas was analysed by energy dispersive X-ray spectroscopy (EDS) (Thermo Fisher Scientific, Waltham, MA, USA). An analysis of the chemical composition within the grains was carried out to determine the content of the individual alloying elements. A minimum of 20 spot analyses were performed in each case. The test was performed at an acceleration voltage of 15 kV. For the selected variants ions, the microstructure of the thin films was examined using a scanning electron microscope equipped with a thin film observation attachment.

The basic mechanical properties: yield strength (YS), ultimate tensile strength (UTS), and percentage elongation (A, %) were determined using an INSPECT 100 tensile testing machine (with a maximum tensile strength of 100 kN). All the tests were performed at least three times.

### 2.5. Optical Scanning of Extruded Tubes and Dies

**Figure 7:**
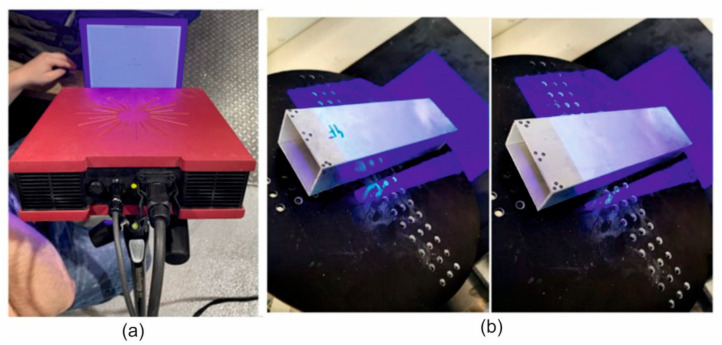
The scanner GOM Atos Core 200 for optically measuring the geometry of the extruded profiles (**a**) and the scanned extruded profiles (**b**).

A GOM Atos Core 200 scanner (Figure 7) was used to scan the extruded elements on the inner and outer surfaces of the samples to measure the wall thickness deviations. The surface was cleaned of impurities prior to the scanning. The coloured map of the deviations was also obtained from the CAD model and the scanned element.

## 3. Results

### 3.1. Microstructural Examination

**Figure 8:**
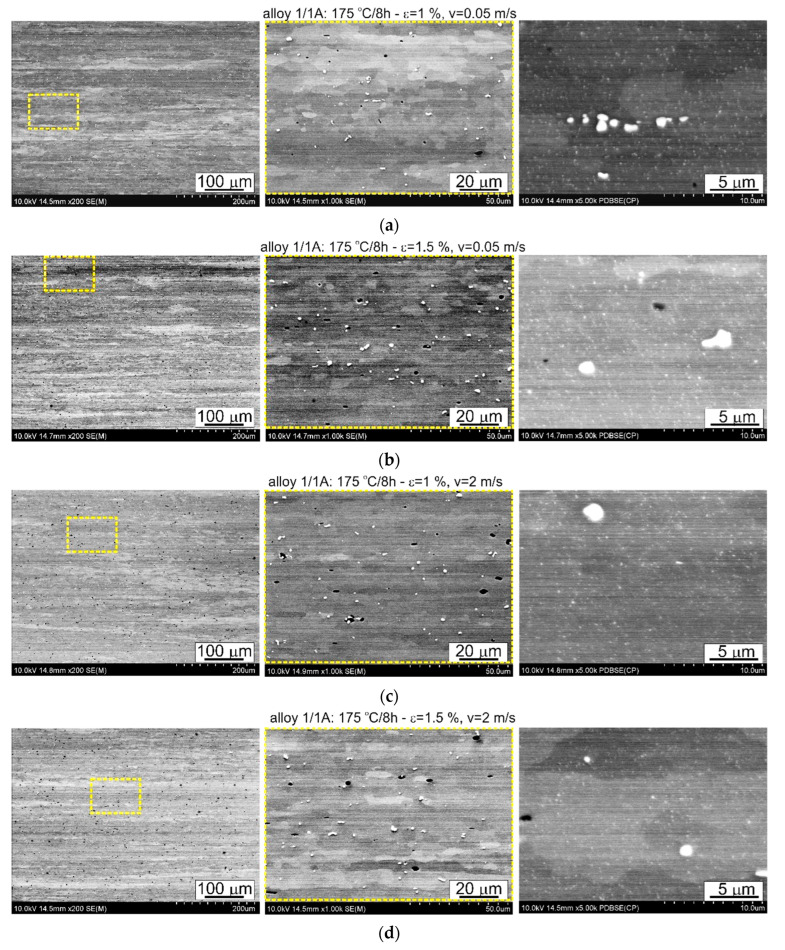
Microstructure of profiles extruded from alloy 1/1A and dynamically straightened at (**a**) ε = 1%, *v* = 0.05 m/s; (**b**) ε = 1.5, *v* = 0.05 m/s; (**c**) ε = 1%, *v* = 2 m/s; (**d**) ε = 1.5%, *v* = 2 m/s; SEM (The middle pictures are enlargements of the area marked by the yellow box in the pictures on the left).

**Figure 9:**
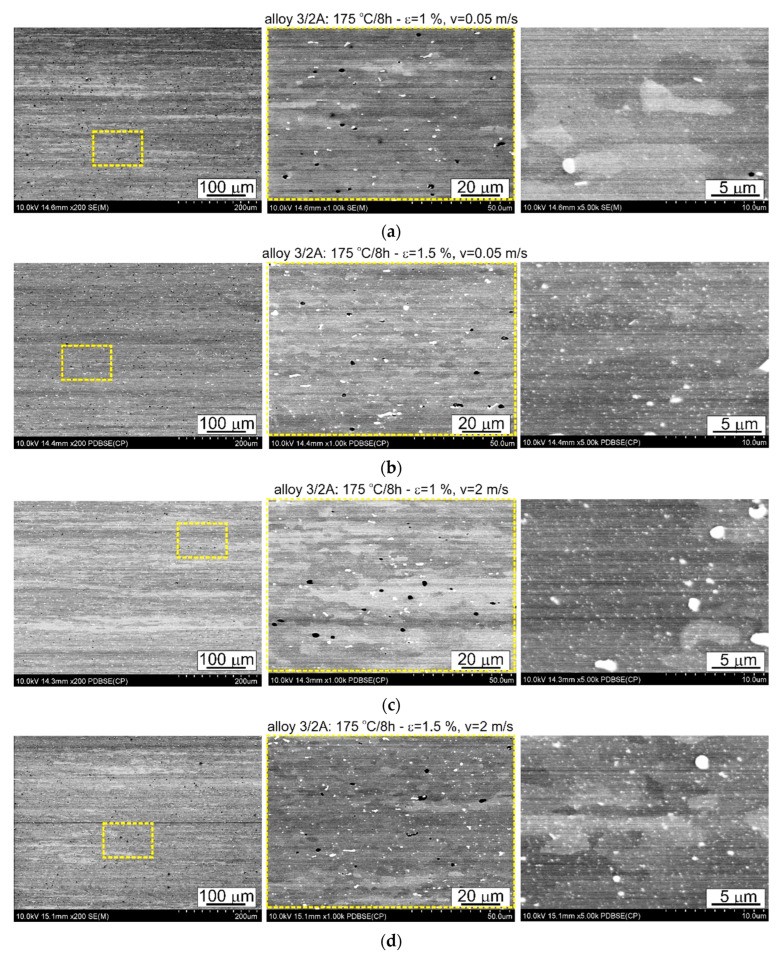
Microstructure of profiles extruded from alloy 3/2A and dynamically straightened at (**a**) ε = 1%, *v* = 0.05 m/s; (**b**) ε = 1.5%, *v* = 0.05 m/s; (**c**) ε = 1%, *v* = 2 m/s; (**d**) ε = 1.5, *v* = 2 m/s; SEM (The middle pictures are enlargements of the area marked by the yellow box in the pictures on the left).

**Figure 10:**
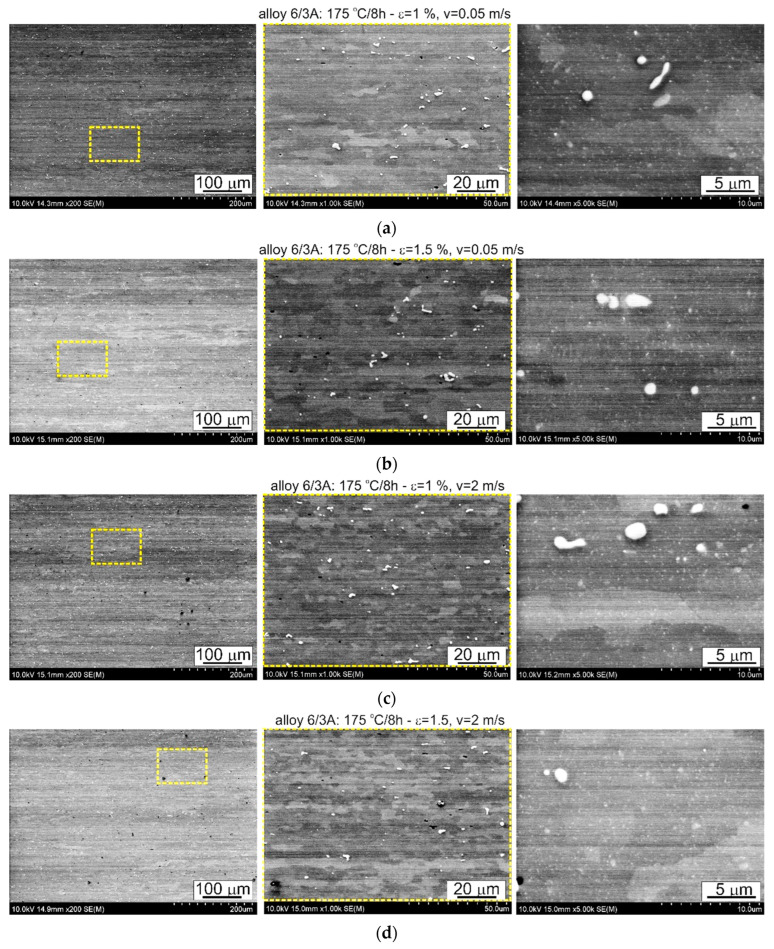
Microstructure of profiles extruded from alloy 6/3a and dynamically straightened at (**a**) ε = 1%, *v* = 0.05 m/s; (**b**) ε = 1.5%, *v* = 0.05 m/s; (**c**) ε = 1%, *v* = 2 m/s; (**d**) ε = 1.5%, *v* = 2 m/s; SEM (The middle pictures are enlargements of the area marked by the yellow box in the pictures on the left).

**Figure 11:**
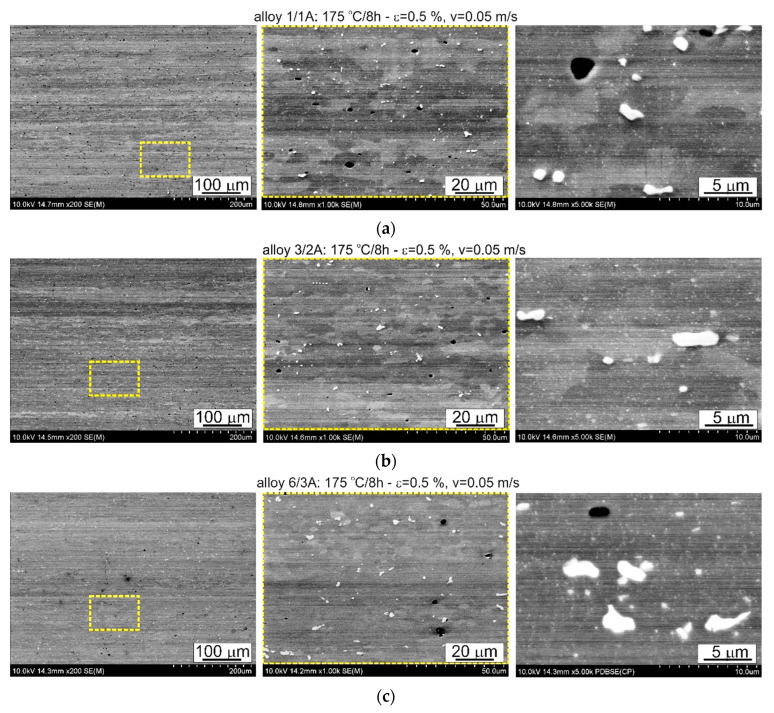
Microstructure of profiles statically straightened at ε = 0.5%, *v* = 0.05 m/s: (**a**) alloy 1/1A, (**b**) alloy 3/2A, and (**c**) alloy 6/3A; SEM (The middle pictures are enlargements of the area marked by the yellow box in the pictures on the left).

**Figure 12:**
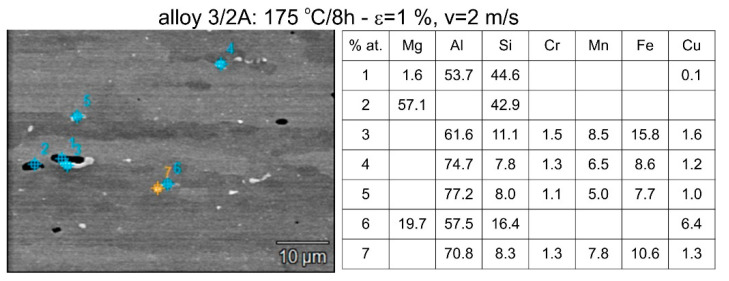
Illustrative outcomes of the chemical analysis depicting the particles within the microstructure of the alloy 3/2A-extruded profiles supersaturated during the press run. Noteworthy particles identified include β-Mg_2_Si, Q-Al_5_Cu_2_Mg_8_Si_6_, and a phase comprising Al, Si, Fe, and Mn.

**Figure 13:**
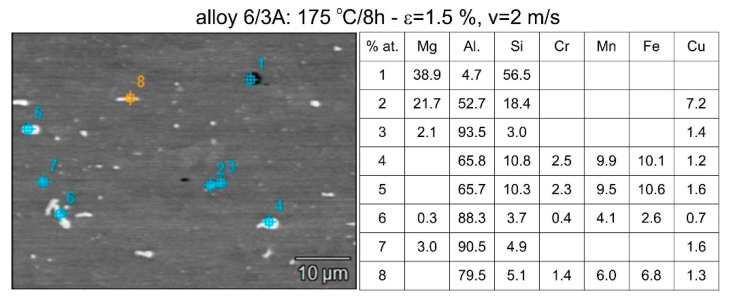
Illustrative outcomes of the chemical analysis depicting the particles within the microstructure of the alloy 6/3A-extruded profiles supersaturated during the press run. Noteworthy particles identified include β-Mg_2_Si, Q-Al_5_Cu_2_Mg_8_Si_6_, and a phase comprising Al, Si, Fe, and Mn.

**Figure 14:**
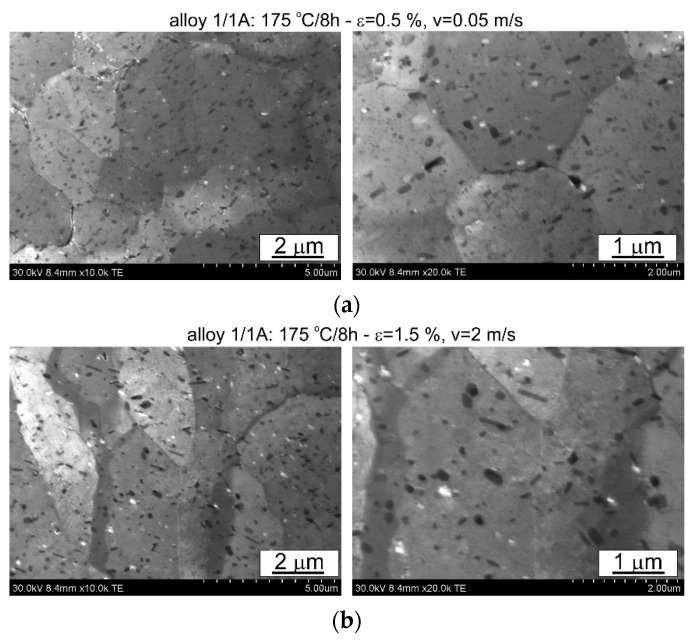
Microstructure of profiles extruded from alloy 1/1A: (**a**) statically straightened at ε = 0.5, v = 0.05 m/s; (**b**) dynamically straightened at ε = 1.5, v = 2 m/s; STEM.

**Figure 15:**
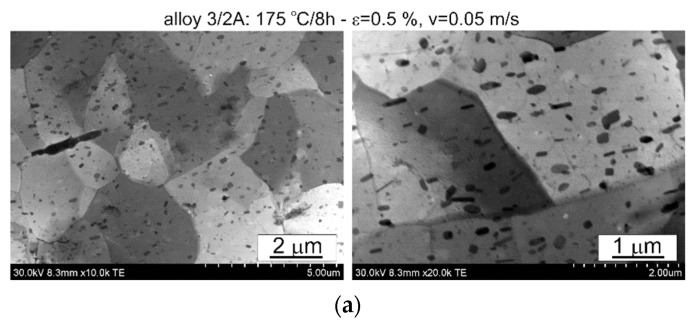

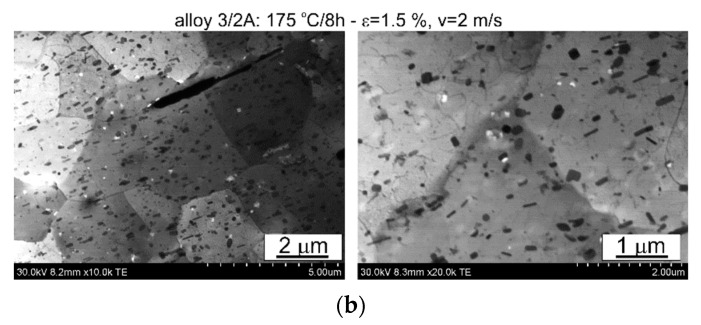
Microstructure of profiles extruded from alloy 3/2A: (**a**) statically straightened at ε = 0.5, *v* = 0.05 m/s; (**b**) dynamically straightened at ε = 1.5, *v* = 2 m/s; STEM.

**Figure 16:**
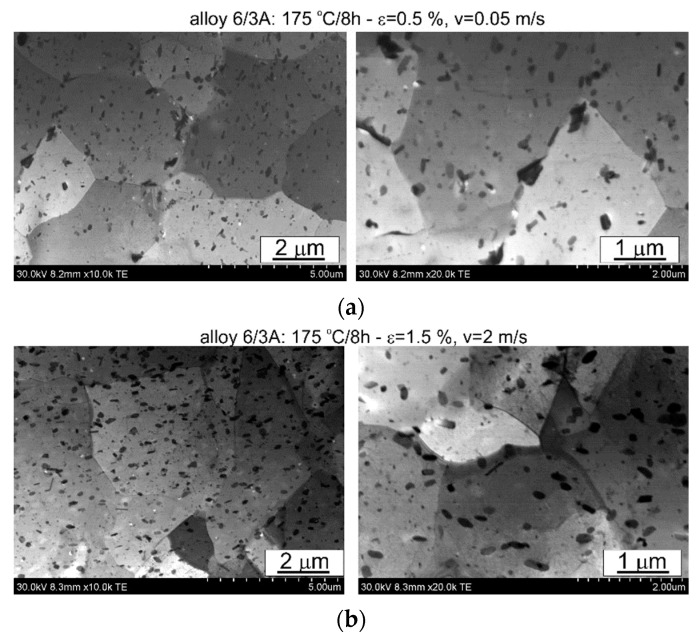
Microstructure of profiles extruded from alloy 6/3A: (**a**) statically straightened at ε = 0.5, v = 0.05 m/s; (**b**) dynamically straightened at ε = 1.5, v = 2 m/s; STEM.

The thermomechanical treatment consisted of straightening the profiles after extrusion, combined with supersaturation during the press run and prior to artificial ageing. The aim of this treatment was to improve the mechanical properties of the profiles. Following the thermomechanical treatment carried out under different conditions (deformation, speed), the ageing process took place at T = 175 °C for t = 8 h. Subsequently, microstructural observations and uniaxial tensile tests were carried out. The representative results of the microstructure tests are shown in Figure 8, Figure 9, Figure 10, Figure 11, Figure 12, Figure 13, Figure 14, Figure 15 and Figure 16 below. These results were obtained after water-cooled extrusion on the press run and the subsequent static or dynamic straightening using the original device designed by the authors. The data are presented as a function of stretch speed and straightening speed.

Differences in the microstructure of the profiles which were tested are revealed by the results of the post-strain microstructure analysis. The microstructure of these profiles typically shows elongated grains which flatten with wall thickness. The character of the grain boundaries varies, taking on a more bulged appearance or remaining flat depending on the specific deformation process variant and alloy chemical composition. Detailed observations at high magnification reveal the presence of near-axial fine grains within the elongated grains, forming bands. The number, shape, and size of these fine grains depend on the parameters of the deformation process, including the deformation size, speed, and whether static or dynamic strain is applied (see Figure 8, Figure 9, Figure 10 and Figure 11). The microstructure of the investigated profiles also contains numerous precipitates. The chemical composition studies using SEM/EDS microarrays confirm the presence of β-Mg_2_Si phase particles up to several μm in size and Q-Al_5_Cu_2_Mg_8_Si_6_ phase particles (see Figure 12 and Figure 13). In addition, advantageous dispersoids are present which improve the mechanical properties of the extruded profiles (see Figure 8, Figure 9, Figure 10 and Figure 11).

For the selected variations, the microstructure of the thin films was examined using a scanning electron microscope equipped with a thin film observation attachment. The representative microstructural images are presented in Figure 14, Figure 15 and Figure 16. The analysis of the thin films revealed that irrespective of the extrusion process parameters applied to the profiles, their microstructure consisted of grains with localised low-energy dislocation systems and a significant presence of precipitates. The quantity and size of these precipitates depend on the alloy composition, with higher alloying elements corresponding to an increase in the quantity and size of the precipitate, as confirmed by the statistical analysis presented later in the article. The low dislocation content is attributed to the presence of microstructural renewal processes during the plastic deformation process. Moreover, the ageing temperature used (175 °C) is sufficient to facilitate microstructural renewal processes. The rearrangement and annihilation of dislocations may have occurred prior to the precipitation of the strengthening phases during the ageing process.

### 3.2. Mechanical Properties

**Figure 17:**
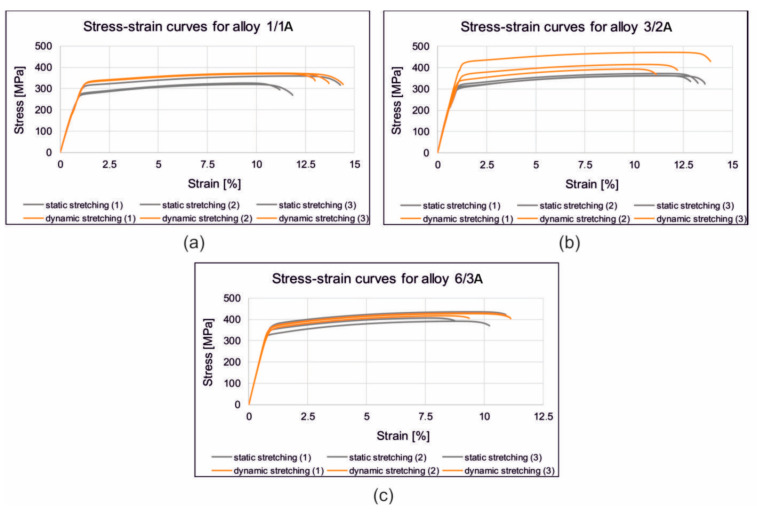
Stress/strain curves recorded during static tensile test of samples from alloys 1/1A (**a**), 3/2A (**b**), and 6/3A-extruded (**c**), statically and dynamically stretched and artificially aged.

**Figure 18:**
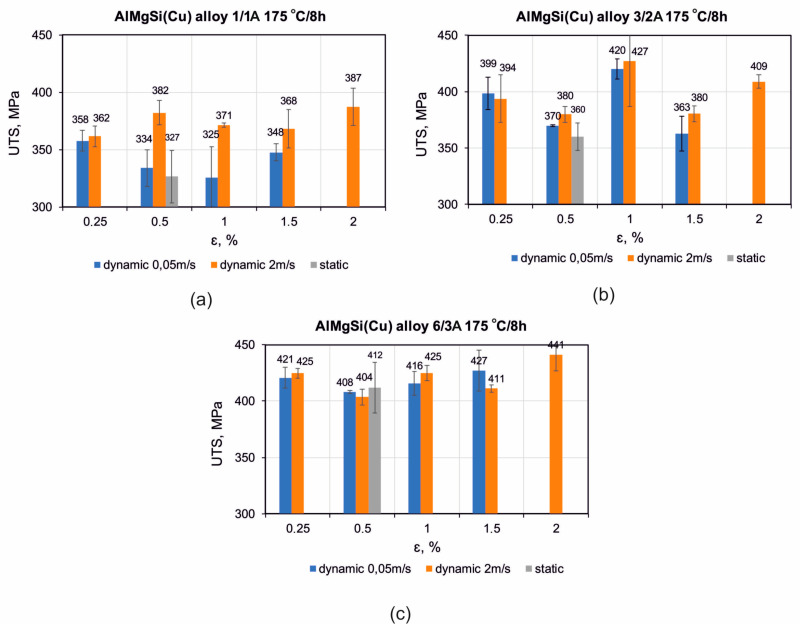
Dependence of tensile strength (UTS) for extruded profiles of alloys (**a**) 1/1A, (**b**) 3/2A, and (**c**) 6/3A, subjected to press run cooling, static or dynamic straightening, and subsequent artificial ageing at 175 °C for 8 h.

Figure 17 shows the stress/strain curves recorded during the static tensile test of the samples from the alloys 1/1A, 3/2A, and 6/3A-extruded, statically and dynamically stretched and artificially aged. Figure 18 shows the correlation between the achieved tensile strength (UTS) of the profiles made from the alloys 1/1A, 3/2A, and 6/3A. The profiles were extrusion-cooled in the press run and then subjected to static or dynamic straightening on the original prototype device. They were then artificially aged (175 °C/8 h). The relationship between the amount of cold deformation and the straightening speed is shown. In particular, there is a significant increase in the tensile strength for the samples subjected to dynamic straightening compared to those straightened statically—approximately an 18% increase for the alloys 1/1A and 3/2A and a 7% increase for alloy 6/3A. In general, slightly higher tensile strength values were observed for the higher stretching speed variant, i.e., 2 m/s, compared to 0.05 m/s. For alloy 1/1A which has the lowest Cu content (0.61%), the highest tensile strength (UTS) was recorded at 2% dynamic deformation, reaching approximately 387 MPa. This contrasts with 326 MPa at 0.5% static deformation. In the case of alloy 3/2A with an intermediate Cu content (0.81%), the maximum tensile strength (UTS) was observed at 1% dynamic deformation which was approximately 432 MPa (compared to 360 MPa for 0.5% static deformation). Similarly, for alloy 6/3A which has the highest Cu content (1.22%), the maximum tensile strength (UTS) was attained with a dynamic deformation of 2%, measuring approximately 441 MPa (as opposed to 412 MPa for a static deformation of 0.5%).

**Figure 19:**
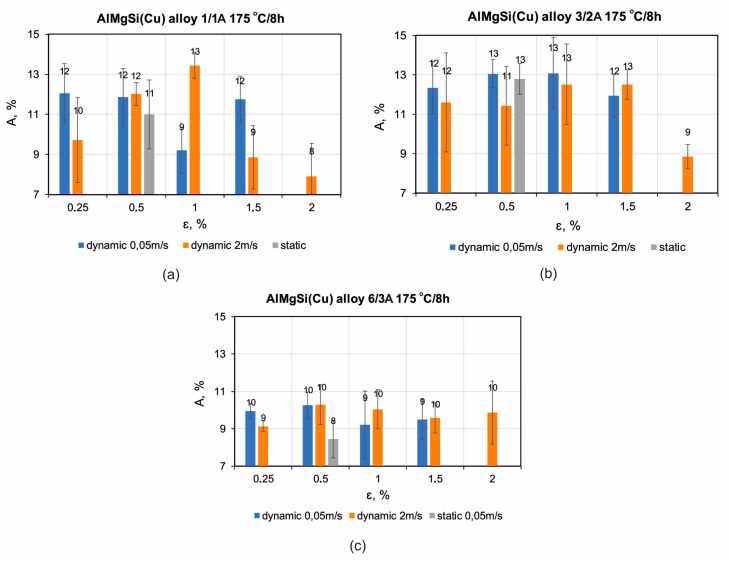
Dependence of elongation (A) for extruded profiles of alloys (**a**) 1/1A, (**b**) 3/2A, and (**c**) 6/3A, subjected to press run cooling, static or dynamic straightening, and subsequent artificial ageing at 175 °C for 8 h.

The elongation value (A, %) for most variants is comparable for the alloys with low and medium Cu content (alloy 1/1A and alloy 3/2A) averaging around 11–13% (Figure 19a,b). For the alloy with the highest Cu content (6/3A), the elongation is lower, at around 9–10% for the samples after the dynamic straightening and 8% for the samples after the static straightening (Figure 19c). These results correlate well with the level of tensile strength, as this alloy has the highest level of mechanical properties.

### 3.3. Geometrical Inspection

**Figure 20:**
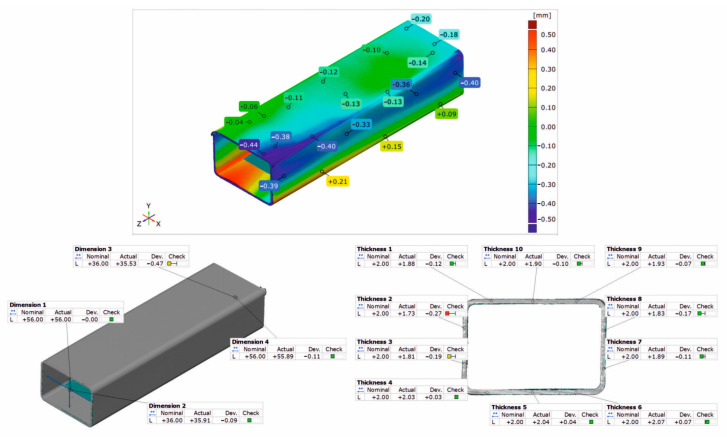
Results of 3D optical scanning of profile of 60 × 40 × 2 mm extruded from alloy 1/1A and dynamically straightened at ε = 2.0, v = 2 m/s.

**Figure 21:**
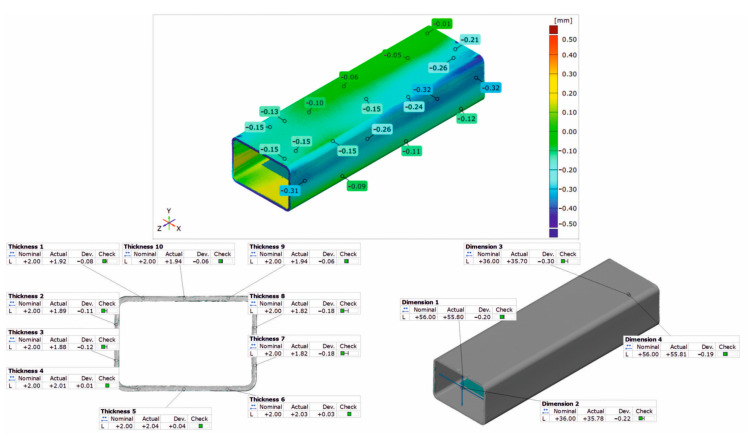
Results of 3D optical scanning of profile of 60 × 40 × 2 mm extruded from alloy 3/2A and dynamically straightened at ε = 2.0, v = 2 m/s.

**Figure 22:**
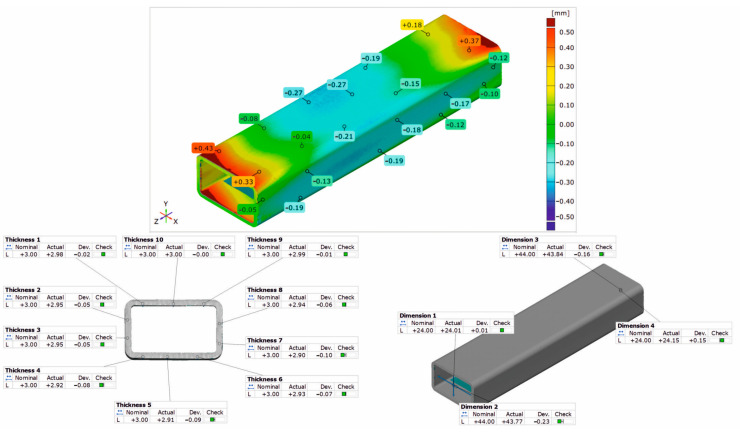
Results of 3D optical scanning of profile of 50 × 30 × 3 mm extruded from alloy 6/3A and dynamically straightened at ε = 2.0, v = 2 m/s.

Figure 20, Figure 21 and Figure 22 show the results of the 3D optical scanning of the profiles extruded from the AlMgSi(Cu) alloys and dynamically stretched with a 2% deformation at a speed of *v* = 2 m/s: the 60 × 40 × 2 mm 1/1A alloy profile (Figure 20), the 60 × 40 × 2 mm 3/2A alloy profile (Figure 21), and the 50 × 30 × 3 mm 6/3A alloy profile (Figure 22). In all the cases, the upper figure shows the coloured map of the dimensional deviations, while the lower figure on the left shows the dimensional deviations of the wall thickness of the profile, and, on the right, the dimensional deviations of the width and height of the profile after extrusion and dynamic stretching. The coloured maps show the relatively large deviations on the longitudinal edge and on the side and bottom walls of the 60 × 40 × 2 mm 1/1A alloy profile (Figure 20). Relatively higher deviations are observed at the ends of the analysed profile, which may be due to the deformation of the ends in the jaws during stretching. However, the most important conclusion concerns the wall thickness, width, and height of the profiles, which are generally within the permissible range of the relevant standard [26]. For all three profiles studied, the acceptable limits of wall thickness deviations according to the standard are in the range of ±0.35 mm and are not exceeded in any case. In the case of the height/width dimensions for the first two profiles, the acceptable limits of deviation are in the range of ±1.00 mm and are also not exceeded, similarly for the 3A alloy, where the acceptable limits of deviation are within the range of ±0.80 mm.

The analysis of the dimensional deviations of the profiles studied shows that they are very accurate. The higher the strength of the alloy (from alloy 1A to alloy 3A), the lower the dimensional deviations of the wall thickness and height/width.

## 4. Discussion

The laboratory dynamic stretching device is relatively simple to operate and maintain. It requires at least two operators working together, who must be trained to carry out the tests. The first operator controls the clamping jaws, the actuator feed, and the tension of the profile for static deformation. The second operator controls the work of the dynamic system. The accuracy of the force measurements is sufficient; for the parameters tested, the load of up to approximately 100 kG on a maximum scale of 50 T represents only about 0.2%. The displacement recorded by a digital straightedge with a reading accuracy of 0.02 mm is sufficient to measure the displacement of the pulling plate and to calculate the deformation. The mechanical system used to clamp the profile in the jaws is sufficient to immobilise it. The set of pneumatic hammers is sufficient to generate the dynamic deformations for the specified cross-section of the profiles. When designing industrial equipment, it is recommended to take into account the digital measurement of static force on hydraulic valves and the measurement of displacement with an accuracy of 0.02 mm. For a higher frequency of impacts, multiple pneumatic hammers and their alternate cyclic work should be applied. If very high deformation and high energy of impacts are required, pneumatic hammers should be replaced by hydraulic cylinders. The cradle for profiles over 15 mm in length should be replaced by the independent traveller with the dynamic set. It is necessary to have a system for damping vibrations and maintaining the initial tension of the profile. A gas spring kit or elastomers can be used. It is recommended to apply the programmer to set the cycle time and the number of repetitions of the hydraulic system. However, it should be remembered that the application of changes and modifications depends on the final design of the device and the assumptions for its use.

The introduction of dynamic stretching facilitates the occurrence of dynamic recrystallisation, a phenomenon which is particularly pronounced in the microstructure of dynamically stretched profiles at ε = 1% and ε = 1.5% with stretching speeds of *v* = 2 m/s. Typically, fine, equiaxial grains are organised in bands, and occasionally, the protrusions of the original grain boundaries are locally visible. Yu et al. [27] observed a similar phenomenon during the porthole die extrusion process of aluminium alloy 6063. Equiaxed grains enter the weld zone and elongate along the extrusion direction under the influence of compression and shear. Subsequently, grain boundaries migrate due to interfacial energy, resulting in grain entrapment and the growth of elongated coarse grains. These elongated coarse grains undergo continuous dynamic recrystallisation and geometric dynamic recrystallisation at high temperatures and pressures, resulting in the formation of fine equiaxed grains.

The profiles subjected to dynamic deformation exhibit superior mechanical properties, especially at higher speeds such as 2 m/s. Consider, for example, the alloy 3/2A profile, where the highest ultimate tensile strength (UTS) is observed at ε = 1%. Compared to both the dynamically and statically stretched profiles, its microstructure appears more homogeneous. Dispersoids are smaller and more abundant, especially in the statically straightened profiles (see Figure 14, Figure 15 and Figure 16). Furthermore, in several cases, particularly with dynamic deformation, the grain boundaries appear to be bulged and numerous fine grains, probably formed during recrystallisation, can be seen in their vicinity.

**Figure 23:**
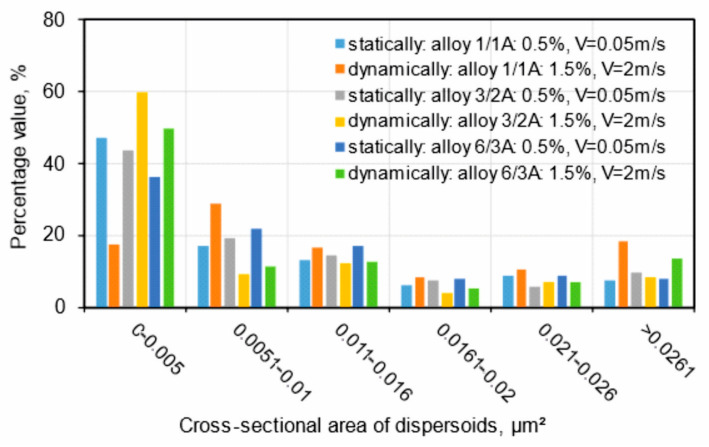
Statistical analysis of dispersoids for extrudates from AlMgSi(Cu) alloys with different Cu contents after static or dynamic stretching.

In order to further analyse the results which were obtained, a statistical analysis of the dispersoids was carried out for the variants from which thin films were made. The free software ImageJ 1.54i was used to determine the cross-sectional area of the dispersoids, and the results are presented graphically in Figure 23 and in Table 4. The results confirmed previous observations regarding the amount of dispersoids as a function of alloy type. The highest amount of dispersoids was found in the alloy with the highest Cu content (alloy 6/3A), occupying 8.1% and 7.7% of the analysed area for the statically and dynamically straightened profiles, respectively. The tensile strength for both the straightening variants was comparable, averaging UTS = 411–412 MPa (Table 3). It is worth noting that for the dynamically straightened alloy 6/3A, 50% of the dispersoids had a cross-sectional area below 0.005 μm^2^, while for the statically straightened variant, 36% of the analysed dispersoid population fell within this range (Figure 23).

For both the alloys with the lowest and the medium Cu content (1/1A and 3/2A alloys, respectively), the total area occupied by dispersoids was greater in the case of static straightening (Table 4). Almost 77% of the dispersoid population measured in the statically straightened profiles of these alloys has a size below 0.016 μm^2^, while for the dynamically straightened profiles, the percentages are 63% and 82%, respectively (Figure 23). The higher level of mechanical properties in the case of the dynamically straightened profiles is mainly due to the size and shape of the grain, which is finer and, depending on the straightening conditions, either elongated in the direction of extrusion or close to equiaxed (Figure 8, Figure 9, Figure 10 and Figure 11).

**Figure 24:**
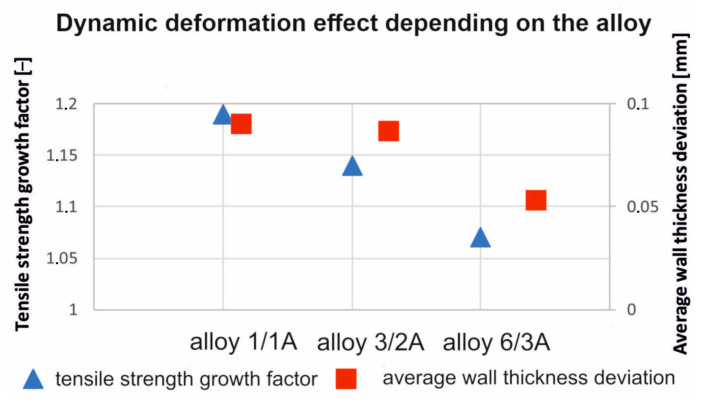
Dynamic deformation effect depending on the alloy for the extruded profiles of the alloys 1/1A, 3/2A, and 6/3A subjected to press run cooling, static or dynamic straightening, and subsequent artificial ageing at 175 °C for 8 h.

Figure 24 shows the effects of applying the dynamic stretching (ε = 2.0, v = 2 m/s) to the extruded profiles of the AlMgSi(Cu) alloys with different alloy components, including Cu. The Cu content is used here as a parameter indicating the increase in the UTS after the dynamic stretching relative to static stretching (left) and as a parameter indicating the average deviation in the wall thickness of the extruded and stretched profiles (right). In both cases, there is a tendency towards failure from the 1/1A alloy with the lowest content of alloying elements to the 6/3A alloy with the highest content of alloying elements. The highest increase in the UTS due to dynamic deformation during the stretching of the profile was obtained for alloy 1/1A (strength factor of 1.18 means an 18% increase in the UTS after dynamic deformation compared to static deformation. Similarly, the highest mean dimensional deviation of wall thickness was observed for this alloy at the level of 0.1 mm (acceptable according to the standard [26]). This is probably due to the finest-grained and most homogeneous microstructure, numerous dispersoids present in this alloy after extrusion, dynamic stretching, and artificial ageing. This alloy, due to the lowest content of alloying elements, is characterised by the lowest yield stress, which translates into the highest dimensional deviations after stretching. For the other alloys (3/2A and 6/3A), dynamic stretching is not so advantageous as the increase in the UTS is only at the level of 13% and 7%, respectively, with respect to the statically (classically) stretched profiles. The 3/2A and 6/3A alloys are characterised by the lower average dimensional deviations of the wall thickness, 0.08 mm and 0.06 mm, respectively, due to the higher content of alloying elements and also to the higher values of the yield stress.

## 5. Conclusions

The following conclusions can be drawn from the results which have been obtained:

1. In this paper, the authors have built a prototype of a device for the dynamic stretching of extruded profiles after quenching. The semi-industrial device is equipped with a hydraulic system for stretching and a pneumatic system for cold dynamic deformation. The aim of this project is to induce advantageous microstructural changes and increase the strength properties of the extruded material. A guideline is formulated for the design of the industrial device in which very long profiles are stretched. This requires the intensification of the dynamic stretching. For high deformations and high impact energy, pneumatic hammers should be replaced by hydraulic actuators.
2. The highest increase in the UTS due to dynamic deformation during the stretching of the profile was obtained for alloy 1/1A (a strength factor of 1.18 means an 18% increase in the UTS after dynamic deformation compared to static deformation). This alloy contains the lowest amount of the alloying elements. For the alloys 3/2A and 6/3A, the dynamic stretching is not so favourable; the increase in the UTS is only 13% and 7%, respectively, with respect to the statically stretched profiles.
3. The application of dynamic stretching to the alloys tested makes it possible to obtain profiles with an advantageous fine-grained microstructure, containing a large number of dispersoids and a homogeneous microstructure, resulting in high strength properties.
4. Dimensional tolerances are not a critical parameter in the dynamic straightening of extruded aluminium profiles with high cold deformation reaching up to 2%. The dimensional tolerances obtained for this dynamic deformation are within the limits of the relevant standard. Therefore, it can be said that the dimensional deviations obtained are not an obstacle to the use of high dynamic deformation in the straightening of extruded aluminium profiles and are advantageous from the point of view of microstructure and mechanical properties.

## Figures and Tables

**Table 1:** Chemical composition of the AlMgSi(Cu) alloys under study, mass per cent.

Alloy Denotation	Si	Fe	Cu	Mg	Cr	Zn	Ti	Zr
AlMgSi(Cu) alloy 1	1.04	0.05	0.61	0.68	0.25	0.01	0.02	0.15
AlMgSi(Cu) alloy 2	1.20	0.04	0.81	0.79	0.23	0.01	0.02	0.15
AlMgSi(Cu) alloy 3	1.21	0.06	1.22	0.80	0.41	0.01	0.02	0.15

**Table 2:** DSC test results of as-cast and homogenised AlMgSi(Cu) alloys.

Alloy	Solidus Temperature, °C	Incipient Melting Heat, J/g
AlMgSi(Cu) alloy 1	544.0	0.61
AlMgSi(Cu) alloy 2	542.8	1.54
AlMgSi(Cu) alloy 3	509.2	0.21
AlMgSi(Cu) alloy 1 (homogenised)	596.1	0.13
AlMgSi(Cu) alloy 2 (homogenised)	584.3	0.32
AlMgSi(Cu) alloy 3 (homogenised)	574.6	1.00

**Table 3:** The parameters of the extrusion process, extrudate stretching, and ageing for the AlMgSi(Cu) alloys of different chemical composition.

Alloy	AlMgSi(Cu) alloy 1, 2 and 3
Billet dimensions	Ø100 × 300 mm
Billet temperature	490 °C
Container/Die temperature	500 °C
Metal exit speed	10 m/min
Extrudates temperature	540 °C
Extrudates length	3600 mm
Stretching strain static	0.5%
Stretching strain dynamic	0.25%, 0.5%, 1%, 1.5% and 2%
Stretching speed	0.05 and 2 m/s
Ageing conditions	175 °C/8 h

**Table 4:** Results of the UTS and cross-sectional area of the dispersoid analysis depending on the alloy and stretching conditions.

	AlMgSi(Cu) Alloy 1	AlMgSi(Cu) Alloy 2	AlMgSi(Cu) Alloy 3
	Percentage value of dispersoids in the investigated area, %		
Statically stretched	7.2	7.5	8.1
Dynamically stretched	5.9	4.4	7.7
	UTS, MPa/Standard deviation, MPa		
Statically stretched	326/23	360/12	412/22
Dynamically stretched	368/17	380/7	411/3

## Data Availability

The original contributions presented in the study are included in the article, further inquiries can be directed to the corresponding author.
